# A SCREEN OF WILD-DERIVED FLY STRAINS REVEALS CG34351 AS A REGULATOR OF TOXIC EFFECTS OF A RICH DIET

**DOI:** 10.1093/geroni/igz038.265

**Published:** 2019-11-08

**Authors:** Rachel Brem

**Affiliations:** Buck Institute for Research on Aging, Novato, California, United States

## Abstract

Dietary restriction (DR) extends lifespan and healthspan, by mechanisms that remain incompletely understood. To identify new genes with a role in dietary response, we developed a screening strategy harnessing genetic variation among wild fruit flies. We quantified the lifespan of lines of the Drosophila Genetic Reference Panel on protein-rich and DR diets, and mapped polymorphisms associated with diet-dependent longevity. Among the hits was CG34351/decima, an ortholog of the mammalian GABA-receptor regulator RGS7BP. In validation experiments, pan-neuronal knockdown of decima extended lifespan and elicited repression of Drosophila insulin-like peptides, in flies on the rich diet but not on DR. Knockdown of decima in GABA neurons rescued fat storage and energy availability defects that derive from overnutrition. We propose that decima tunes signaling in these neurons upstream of the insulin pathway to rewire metabolism. These results establish decima as a determinant of the toxic effects of the rich diet in the fly.

